# Supplementary material for: New Series of Red-Light Phosphor Ca9−xZnxGd0.9(PO4)7:0.1Eu3+ (x = 0–1)
Source: Molecules. 2023 Jan 1;28(1):352. doi: 10.3390/molecules28010352 (PMC9822231; doi:10.3390/molecules28010352)
Supplement: Supplementary file 1 [file molecules-28-00352-s001.zip › molecules-2104332-supplementary.pdf]

Supplementary information

New Series of Red-Light Phosphor $Ca_{9-x}Zn_xGd_{0.9}(PO_4)_7$:$0.1Eu^{3+}$ (x = 0–1)

Yury Yu. Dikhtyar [1], Dmitry A. Spassky [2,3,*], Vladimir A. Morozov [1], Sergey N. Polyakov [4], Valerya D. Romanova [1], Sergey Yu. Stefanovich [1], Dina V. Deyneko [1,5], Oksana V. Baryshnikova [1], Ivan V. Nikiforov [1] and Bogan I. Lazoryak [1]

Table S1. The unit cells parameters, SHG signals ($I_{2\omega}/I_{2\omega}(SiO_2)$) and supposed SG for $Ca_{9-x}Zn_xGd(PO_4)_7$:$0.1Eu^{3+}$ compounds.

x	a, Å	c, Å	V, Å^3	$I_{2\omega}/I_{2\omega}(SiO_2)$	Supposed space group
0.00	10.4489(4)	37.459(3)	3541.8(3)	1.10(5)	
0.05	10.4537(4)	37.481(3)	3547.2(3)	1.05(5)	
0.10	10.4487(4)	37.459(3)	3541.7(3)	1.00(5)	
0.15	10.4419(4)	37.444(3)	3535.7(3)	0.95(5)	$R3c$
0.20	10.4431(4)	37.468(3)	3538.8(4)	1.05(5)	
0.25	10.4398(8)	37.451(5)	3534.9(6)	0.95(5)	
0.30	10.4387(4)	37.448(3)	3533.8(4)	0.95(5)	
0.35	10.4346(4)	37.444(3)	3530.7(3)	0.90(5)	
0.40	10.4298(4)	37.423(3)	3525.5(4)	0.70(5)	
0.45	10.4182(4)	37.396(3)	3515.1(3)	0.50(5)	
0.50	10.3980(4)	37.278(3)	3490.4(3)	0.45(5)	
0.55	10.3913(3)	37.263(2)	3484.0(3)	0.30(5)	$R3c + R\bar{3}c$
0.60	10.3880(4)	37.250(3)	3481.2(3)	0.25(5)	
0.65	10.3772(3)	37.184(2)	3468.1(2)	0.20(5)	
0.70	10.3720(5)	37.080(2)	3440.9(3)	0.10(5)	
0.75	10.3700(6)	37.150(2)	3459.8(3)	0.07(5)	

0.80	10.3680(7)	37.145(3)	3458.0(4)	0.06(5)	
0.85	10.3641(7)	37.132(3)	3454.1(4)	0.00(5)	
0.90	10.3609(7)	37.116(3)	3450.6(4)	0.00(5)	$R\bar{3}c$
0.95	10.3596(7)	37.113(3)	3449.3(4)	0.00(5)	
1.00	10.3596(6)	37.100(2)	3448.2(3)	0.00(5)	

Table S2. Fractional atomic coordinates, site symmetry, isotropic displacement atomic parameters (U_{iso}) and site occupation for $Ca_8ZnGd(PO_4)_7$ from PXRD data.

Atom	Site	*x*	*y*	*z*	U_{iso}×100	*Occupancy* a_i, M
*M*1	36*f*	0.7230(1)	0.8511(3)	0.4342(1)	0.36(4)	0.8770(1) Ca^{2+}<br>0.1230(2) Gd^{3+}
*M*3	36*f*	0.1356(9)	0.2750(6)	0.3264(1)	2.88(3)	0.4576 Ca^{2+}<br>0.0424 Gd^{3+}
*M*5	6*b*	0	0	0	0.05(1)	Zn^{2+}
P1	12*c*	0	0	0.2687(3)	0.01(1)	0.5P
P2	36*f*	0.3174(3)	0.1741(5)	0.3644(1)	0.14(2)	P
O1	12*c*	0	0	0.3113(1)	0.64(1)	0.5O
O2	36*f*	0.0097(2)	0.8592(2)	0.2600(4)	0.64(1)	0.5O
O3	36*f*	0.2793(6)	0.1683(8)	0.3237(2)	0.64(1)	O
O4	36*f*	0.2474(9)	0.0157(9)	0.3840(2)	0.64(1)	O
O5	36*f*	0.2722(9)	0.2871(9)	0.3826(2)	0.64(1)	O
O6	36*f*	0.4978(7)	0.2528(2)	0.3690(1)	0.64(1)	O

Table S3. Selected distances (Å) in $Ca_8ZnGd(PO_4)_7$ from PXRD data.

Polyhedron	Distance	*d*, Å	Polyhedron	Distance	*d*, Å
$M1O_9$	*M*1-O2	2.447(9)	$M3O_8$	*M*3-O1	2.534(5)
	*M*1-O2	2.366(9)		*M*3-O2	2.796(2)
	*M*1-O3	2.318(7)		*M*3-O3	2.261(9)
	*M*1-O4	2.283(9)		*M*3-O3	2.735(9)
	*M*1-O4	2.679(9)		*M*3-O4	2.558(9)
	*M*1-O5	2.399(9)		*M*3-O4	2.573(9)
	*M*1-O5	2.552(9)		*M*3-O5	2.492(9)
	*M*1-O6	2.423(9)		*M*3-O5	2.255(9)
	*M*1-O6	2.468(9)		**<*M*3-O>**	2.526(8)
	**<*M*1-O>**	2.437(9)			
$M5O_6$	6×*M*5-O6	2.014(6)	$P2O_4$	P2-O3	1.555(7)
$P1O_4$	P1-O1	1.582(9)		P2-O4	1.601(9)
	3×P1-O2	1.547(9)		P2-O5	1.612(9)
				P2-O6	1.634(7)
	**<P1-O>**	1.556(9)		**<P2-O>**	1.601(8)
